# Genomic and structural analysis of the endocannabinoid system in Moroccans: A novel CNR1 variant (V392A) impairs CB1 receptor stability

**DOI:** 10.1371/journal.pone.0347606

**Published:** 2026-04-30

**Authors:** Hanane Abbou, Rihab Festali, Mohammed Walid Chemao-Elfihri, Mohammed Hakmi, Souad Kartti, Saber Boutayeb, Lahcen Belyamani, Rachid Eljaoudi

**Affiliations:** 1 Laboratory of Drug Sciences (LRSM), Mohammed VI Faculty of Medicine, Casablanca, Mohammed VI University of Sciences and Health (UM6SS), Casablanca, Morocco; 2 Mohammed VI Center for Research and Innovation (CM6RI), Casablanca, Morocco; 3 Research Laboratory of Microbiology, Infectious Diseases, Allergology and Pathogen Surveillance (LARMIAS), Mohammed VI Faculty of Medicine, Mohammed VI University of Sciences and Health (UM6SS), Casablanca, Morocco; 4 Mohammed VI University of Sciences and Health (UM6SS), Casablanca, Morocco; 5 Biotechnology lab (MedBiotech), Bioinova Research Center, Medical and Pharmacy School, Mohammed V University, Rabat, Morocco; UMR-S1134, INSERM, Université Paris Diderot, INTS, FRANCE

## Abstract

The Endocannabinoid System (ECS) is vital for human physiology, and genetic variation within its components can alter function and therapeutic responses. The genetically diverse Moroccan population offers a unique opportunity for pharmacogenomic insight. This study performs a comprehensive genomic analysis of 11 core ECS genes in 109 Moroccan individuals, identifying 170 novel variants. We then performed a deep *in silico* functional characterization of a novel, high-confidence *CNR1* variant (V392A). Extensive, triplicate 500 ns molecular dynamics (MD) simulations were conducted on both the wild-type (WT) and mutant (MT) CB1 receptor. The simulations revealed that the V392A mutation induces significant structural destabilization, evidenced by increased RMSD/RMSF, a consistent loss of α-helicity, and altered interhelical distances crucial for G-protein coupling. Free Energy Landscape (FEL) analysis confirmed the mutant receptor occupies a broader, higher-energy conformational state. This “genomics-to-function” pipeline demonstrates that the Moroccan population harbors significant novel ECS genetic diversity and that the unique V392A *CNR1* variant impairs CB1 receptor stability, suggesting a potential for altered signaling and pharmacological responses.

## 1. Introduction

The Endocannabinoid System (ECS) is a complex molecular and biological system discovered in 1988 by scientists Allyn Howlett and W.A. Devane, who first described the CB1 receptor [1,2]. The word “Endocannabinoid” was first coined after the discovery of a membrane receptor, which would later be recognized as CB1, encoded by the gene *CNR1* [3]. The ECS plays major roles in multiple physiological processes, including homeostasis, emotional and neurological processes, fertility, pregnancy, and pre- and post-natal development [4–6].

Lu and Mackie described the ECS as a widespread neuromodulatory network that plays a crucial role in the development of the CNS and in regulating its responses to both internal and external stimuli. The ECS is composed of cannabinoid receptors (CB1 and CB2), as well as endogenous ligands known as endocannabinoids (eCB), including anandamide (AEA) and 2-arachidonoylglycerol (2-AG), which are the most extensively studied [7,8]. Along with their synthesis and degradation enzymes, all work together to maintain neural homeostasis and modulate physiological processes [9]. AEA is synthesized by N-acyl phosphatidylethanolamine phospholipase D (NAPE-PLD), while 2-AG is a product of hydrolysis of membrane-derived diacylglycerol by sn1-diacylglycerol lipase (DAGL) [10–13]. As for their degradation, AEA is catabolized by Fatty acid amide hydrolase (FAAH), while 2-AG is preferentially degraded through hydrolysis by monoacylglycerol lipase (MAGL) [14–17].

Understanding the intricate signaling mechanisms of the ECS is essential for appreciating how genetic variation may modulate its function and pharmacological responsiveness [5]. Variants in these core genes can influence receptor binding affinity, enzymatic activity, or downstream signaling pathways, ultimately altering individual responses to cannabinoid-based therapies.

In this context, Morocco provides a unique opportunity for genomic exploration, as its population is shaped by a complex history of admixture between Berber, Arab, Sub-Saharan, and Mediterranean ancestries, resulting in a rich genetic mosaic. Moreover, identified Moroccans as a valuable reference for exploring region-specific alleles that may affect ECS-related functions [18].

Leveraging data from the Moroccan Genome Project (MGP), this study presents a comprehensive analysis of the genetic variants found within core ECS genes, including the cannabinoid receptors (*CNR1, CNR2*), and metabolic enzymes (*FAAH, MGLL, DAGLA, DAGLB, NAPEPLD, ABHD6, ABHD12*), and auxiliary components (*TRPV1, GPR55*).

### Specific focus: structural and functional characterization

Our genomic screening identified a novel missense variant, V392A, in the CB1 receptor. Given that functional prediction tools consistently suggested this variant could severely alter protein function, and because of its critical location in the transmembrane domain 7 (TM7), this study places a specific focus on the structural and functional effect of this mutation on the CB1 receptor.

We employ a computational biophysics approach centered on molecular dynamics (MD) simulations to move beyond sequence-based prediction. This structural analysis allows us to describe, at the atomic level, the potential consequences of a novel, population-specific variant on CB1 receptor flexibility, stability, and signal transduction pathways. This pipeline allows for the functional characterization of the V392A CB1 receptor, thereby transforming genomic variant identification into actionable mechanistic insights.

## 2. Methods

### 2.1. Data source

This retrospective study utilized previously published genomic data from MGP [18]. We received access to the data on April 11, 2025, through their online database (http://www.mvv.cm6ri.ma/), after requesting access via the European Genome-phenome Archive (EGA) using the study ID (EGAC50000000353). The accessed data had no information that could identify individual participants during or after data collection.

The previous study utilized the Illumina NovaSeq 6000 platform to generate 150 bp paired-end reads to a minimum depth of 30 × . After initial variant calling, the GATK Variant Quality Score Recalibration (VQSR) filter was applied to ensure high-confidence genotypes. The resulting multi-sample VCF was then processed using BCFtools v1.15.1 to split multi-allelic sites and normalize indels. Subsequent quality control was performed in PLINK2, where variants with over 10% missing genotypes, variants on the Y and mitochondrial chromosomes, and those deviating from Hardy-Weinberg equilibrium (p < 5e − 07) were excluded. Finally, the filtered variants were annotated against the GRCh38/hg38 reference genome using Ensembl VEP v113.0, with default parameters and a custom flag to prioritize pathogenicity scores from ClinVar 2025 [18].

### 2.2. Variant selection and classification

Variants were selected from ECS genes, including *CNR1, CNR2, FAAH, MGLL, DAGLA, DAGLB, NAPEPLD, ABHD6, ABHD12, TRPV1,* and *GPR55*, to capture the full spectrum of canonical and associated endocannabinoid signaling components [19].

Each variant was classified according to its functional impact, based on VEP-defined categories: high impact for variants predicted to cause disruptive effects on protein function or structure, moderate impact for variants expected to disrupt protein function without causing complete loss, low impact for those predicted to have minimal effect on protein structure or activity, and modifier for variants located in non-coding regions or with unknown functional effects.

The functional impact predictors SIFT [20], PolyPhen-2 [21], and AlphaMissense [22] were specifically used to assess the potential effects of missense variants on the protein encoded by each gene.

### 2.3. Computational evaluation of the high-confidence, novel V392A variant in the CNR1 gene

Based on the variant impact assessment, a novel missense variant in the CB1 protein (CNR1 gene) was identified. This variant was selected for further computational evaluation because of its novelty, as it was not reported in any global population database, including gnomAD, 1000 Genomes, or dbSNP, making it unique to the Moroccan cohort, and functional prediction tools used in this study consistently suggested this mutation could alter protein structure and function. Additionally, CB1 is the main cannabinoid receptor in the brain, and the location of the mutation (position 392) is essential for receptor flexibility and G-protein coupling, potentially altering signal transduction [23].

Given these factors, this mutation was prioritized for molecular dynamics simulations to evaluate its structural and functional effects relative to the wild-type receptor. This approach allows us to investigate the potential consequences of a novel, population-specific variant on CB1 receptor dynamics and activity.

#### 2.3.1. Residue conservation analysis.

A residue conservation analysis was conducted to assess the importance of the mutation’s position. According to conservation principles, the more conserved a residue is, the more important it is for proper protein folding and functionality [24]. We retrieved the CB1 sequence from UniProt (P21554) [24] and uploaded it to the Blastp online server [25] using the Reference protein database. From the top 100 results, low-quality proteins, predicted proteins, and additional isoforms were removed. Clustal Omega [25] was used for sequence alignment, and the resulting file was then uploaded to WebLogo [26] to generate a graphical representation of residue variation at each position. Conserved residues are shown using the one-letter amino acid code, while non-conserved positions display the one-letter codes of all amino acids observed at that site in the aligned sequences.

#### 2.3.2. Protein preparation.

The inactive CB1 structure was retrieved from the Protein Data Bank [27] (PDB ID: 5u09 [28]). This structure was chosen as it is the most complete X-ray structure. This 5u09 structure had 1 mutation, T210A, and reverted to the wildtype form using the mutagenesis tool in Maestro. Also, the ICL3 loop was missing and modeled using the Schrödinger Prime module [13]. The final model was minimized and optimized for subsequent analyses. The quality of the newly generated model was assessed using SAVES 6.0, through its tool PROCHECK [26,27].

After model validation, it was used as a template to generate the variant V392A (MT) using Maestro [28].

#### 2.3.3. MD simulation.

Molecular dynamics (MD) simulations of the wild-type (WT) and V392 mutant (MT) CB1 receptor were performed using Desmond, using OPLS4 force field [29]. Each system was embedded in a POPC (1-palmitoyl-2-oleoyl-sn-glycero-3-phosphocholine) lipid bilayer, aligned according to OPM (Orientations of Proteins in Membranes) coordinates for CB1, inside a TIP3P water box [30], neutralized with counter ions (Na+ and Cl⁻), and supplemented with 0.15 M NaCl, in an orthorhombic box with a 10 Å buffer. Before the production run, the system underwent a multistage equilibration protocol, including Brownian dynamics NVT simulation at 10 K (50 ps) with restraints on solute heavy atoms, NVT and NPgT simulations at 100 K, heating from 100 K to 300 K over 150 ps, and a final relaxation step at 300 K.

Each system was simulated for 500 ns across three independent replicas, each initialized with a different random seed (S1 Supplementary, S2 Supplementary, and S3 Supplementary). Short-range interactions were treated with a 9 Å cutoff, and trajectories were saved every 100 ps with periodicity corrections. Energies and simulation box dimensions were recorded at regular intervals. No restraints were applied, and analyses focused on Root Mean Square Deviation (RMSD), Root Mean Square fluctuation (RMSF), Secondary Structure Evolution (SSE), specific transmembrane helix (TM) distances: TM3 (R214) to TM6 (D338) and TM3 (R214) to TM7 (D403), and intramolecular H-Bonds, using Maestro’s Simulation Interaction Diagram (SID) and Simulation Event Analysis (SEA) tools to evaluate receptor dynamics.

Free energy landscapes (FELs) were generated from the six trajectories (three for the WT structure and three for the MT structure), using the MDAnalysis [31,32] Python package. Backbone RMSD and radius of gyration (Rg) were calculated as collective variables and used to construct two-dimensional histograms with consistent binning across all replicas. Free energy surfaces were obtained by converting population densities into free energy values, normalized to the global minimum, and visualized as combined 2D contour maps and 3D projections with a common color scale for WT and MT. The most representative conformations were extracted from the global energy minima of both the WT and MT landscapes to visualize the significant structural shifts caused by the mutation.

## 3. Results

### Part 1: Genomic landscape of the Moroccan ECS cohort

**Fig 1:**
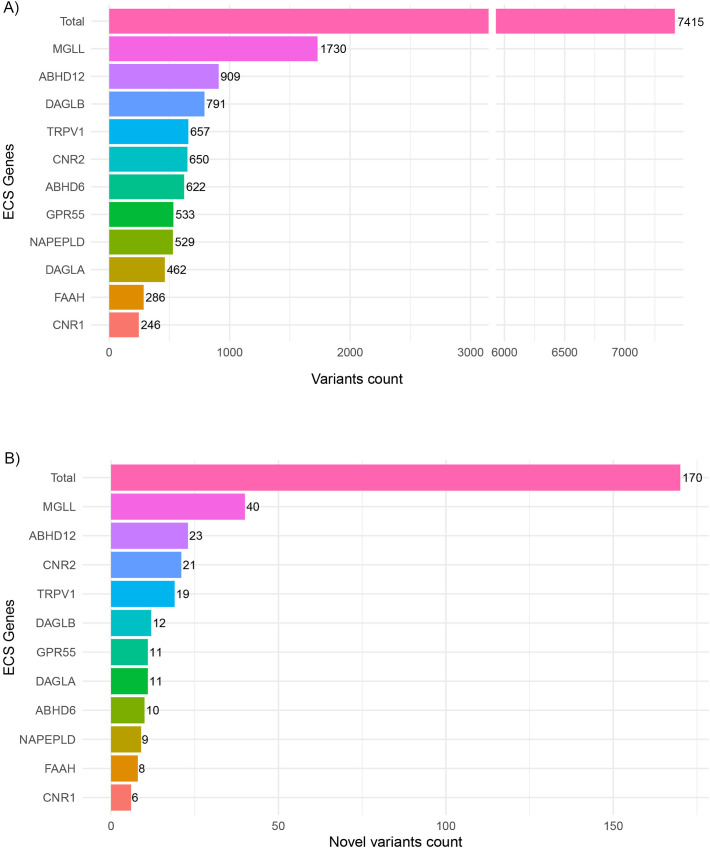
Distribution of genetic variants in eleven ECS genes from the Moroccan study population, showing A) all identified variants and B) novel variants.

We analyzed genomic variations across eleven genes of the ECS in 109 Moroccan individuals. These genes included canonical receptors (*CNR1, CNR2*), biosynthetic and degradative enzymes (*DAGLA, DAGLB, MGLL, FAAH, ABHD6, ABHD12, NAPEPLD*), and non-canonical receptors (*TRPV1, GPR55*). A total of 7415 variants were identified across all ECS genes, with gene-specific counts ranging from 246 in the *CNR1* gene up to 1730 in the *MGLL* gene (Fig 1-A, S1 Supplementary).

From the 7415 variants identified, 170 were novel. These variants were defined by the absence of any recorded identifier in public databases and the absence of a known allele frequency across all global populations. The majority of novel variants were located in *MGLL*, *ABHD12, CNR2*, and *TRPV1*(Fig 1-B, S1 Supplementary).

The variant impact analysis revealed the presence of a high percentage of modifier variants (98.6%), followed by low-impact variants (0.82%), and moderate-impact variants (0.58%). We found no “high-impact” mutations within the ECS genes in our study population (Table 1).

**Table 1:** VEP impact classification of genetic variants identified in ECS genes among the Moroccan population.

Gene	High Impact	Moderate Impact	Low Impact	Modifier Impact
ABHD12	0	1	1	907
ABHD6	0	0	0	622
CNR1	0	2	4	240
CNR2	0	4	8	638
DAGLA	0	3	7	452
DAGLB	0	10	13	768
FAAH	0	4	8	274
GPR55	0	4	3	526
MGLL	0	1	3	1726
NAPEPLD	0	2	6	523
TRPV1	0	12	8	637
Total Count	0	43	59	7313

Out of the 7415 identified variants, 7324 are located in non-coding regions. Only the exonic variants (91 variants) were considered for further investigation.

Among the exonic variants, we identified 48 low-impact synonymous and 43 moderate-impact missense variants, two of which were novel. The majority (27.9%) were located in the *TRPV1* gene, and *ABHD12* and *MGLL* each harbored only one missense variant. Variants were considered pathogenic when all three prediction tools classified them as such (Table 2).

**Table 2:** Mutational pathogenic impact of missense mutations in the Moroccan study population. Pathogenicity was predicted using SIFT, PolyPhen-2, and AlphaMissense. Values shown in parentheses () indicate the raw prediction scores from these tools.

Gene	dbSNP ID	Residue substitution	SIFT	Polyphen-2	AlphaMissense
ABHD12	rs147070618	D135E	tolerated(0.05)	benign(0.005)	likely benign
**CNR1**	**NA**	**V392A**	**deleterious(0)**	**possibly damaging(0.785)**	**likely pathogenic**
CNR1	rs772492894	A446T	tolerated(0.08)	benign(0.037)	likely benign
CNR2	rs148656213	R307H	deleterious(0.03)	benign(0.107)	likely benign
CNR2	rs142861964	A199T	tolerated(0.36)	benign(0.021)	likely benign
CNR2	rs2501432	Q63R	tolerated(1)	benign(0)	likely benign
CNR2	rs2229579	H316Y	tolerated(1)	benign(0.06)	likely benign
DAGLA	rs35056845	G735V	deleterious(0.01)	probably damaging(0.998)	likely benign
DAGLA	rs149360213	A749V	deleterious(0.04)	possibly damaging(0.494)	likely benign
DAGLA	rs3741252	P889L	tolerated low confidence(0.22)	benign(0)	likely benign
**DAGLB**	**rs199661837**	**V231A**	**deleterious(0)**	**possibly damaging(0.806)**	**likely_pathogenic**
DAGLB	rs1055430	L456V	deleterious(0)	possibly damaging(0.852)	likely benign
**DAGLB**	**rs139753251**	**I596T**	**deleterious(0)**	**possibly damaging(0.908)**	**likely pathogenic**
**DAGLB**	**rs145491977**	**T84R**	**deleterious(0)**	**probably damaging(0.997)**	**likely pathogenic**
DAGLB	rs138713047	R646W	deleterious(0.01)	possibly damaging(0.645)	likely benign
DAGLB	rs1133850	A517V	tolerated(0.11)	benign(0.066)	likely benign
DAGLB	rs757204352	F221L	tolerated(0.45)	benign(0.011)	ambiguous
DAGLB	rs117103601	A103V	tolerated(0.69)	benign(0.003)	likely benign
DAGLB	rs116243689	V659I	tolerated(0.74)	benign(0)	likely benign
DAGLB	rs2303361	Q664R	tolerated low confidence(0.29)	benign(0)	likely benign
FAAH	rs77101686	A356V	deleterious(0)	probably damaging(0.935)	likely benign
FAAH	rs200172860	T511M	tolerated(0.09)	benign(0.112)	likely benign
FAAH	rs324420	P129T	tolerated(0.12)	benign(0.018)	likely benign
FAAH	rs75429705	A476G	tolerated(1)	benign(0)	likely benign
GPR55	NA	I292N	tolerated(0.05)	benign(0.001)	ambiguous
GPR55	rs3749073	G195V	tolerated(0.3)	benign(0.036)	likely benign
GPR55	rs80290067	V103I	tolerated(0.47)	benign(0.013)	likely benign
GPR55	rs767722127	R318Q	tolerated low confidence(0.07)	possibly damaging(0.66)	likely benign
MGLL	rs201175620	S16F	deleterious(0)	benign(0.36)	likely benign
NAPEPLD	rs12540583	S152A	tolerated(0.13)	benign(0.001)	likely benign
NAPEPLD	rs3181009	D389N	tolerated(0.44)	benign(0.001)	likely benign
TRPV1	rs224534	T469I	deleterious(0.01)	benign(0.058)	likely benign
TRPV1	rs199705101	S55L	deleterious(0.04)	benign(0)	likely benign
TRPV1	rs200296818	G470R	tolerated(0.1)	benign(0.025)	likely benign
TRPV1	rs199726079	A280T	tolerated(0.12)	benign(0.326)	likely benign
TRPV1	rs55916885	Q85R	tolerated(0.22)	benign(0)	likely benign
TRPV1	rs8065080	I585V	tolerated(0.35)	benign(0)	likely benign
TRPV1	rs222749	P91S	tolerated(0.73)	benign(0.054)	likely benign
TRPV1	rs202106784	S510G	tolerated(0.92)	benign(0.015)	likely benign
TRPV1	rs222747	M315I	tolerated(1)	benign(0)	likely benign
TRPV1	rs200164496	A13V	tolerated low confidence(0.08)	benign(0.001)	likely benign
TRPV1	rs146183848	S821F	tolerated low confidence(0.25)	possibly damaging(0.453)	likely benign
TRPV1	rs146598604	L9F	tolerated low confidence(0.72)	benign(0.129)	likely benign

**Fig 2:**
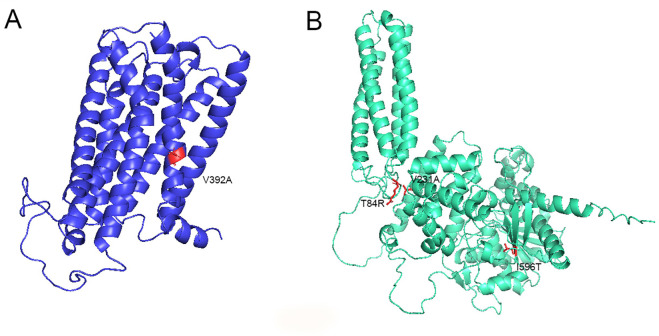
3D structures ofA) CB1 receptor and B) DAGLB enzyme (AlphaFold entry: AF-Q8NCG7-F1), with pathogenic variants identified in the Moroccan study population highlighted in red (one on CB1, three on DAGLB).

Of the 43 missense mutations, 4 have been identified as pathogenic, based on consistent results from mutational effect assessors used in this study. Three of these mutations occurred in the *DAGLB* gene, and one in the CB1 gene. Additionally, the CB1 mutation V392A emerged as a novel variant (Fig 2).

### Part 2: In silico functional characterization of the novel CNR1-V392A variant

Analysis of WGS data from the 109-individual Moroccan cohort identified a novel, heterozygous variant in the CNR1 gene at position chr6:88144100. This variant call was supported by robust WGS coverage, with a total depth of 3926 reads across the 109-sample cohort (average depth of approximately 36). The variant was observed as a singleton, with a maximum likelihood allele count of 1 out of 218 total alleles (MLEAC = 1, AN = 218), corresponding to a rare allele frequency of 0.0046 (0.46%) in this population. The V392A variant was not reported in any global population database, including gnomAD, 1000 Genomes, or dbSNP, making it unique to the Moroccan cohort, and functional prediction tools confirmed its potential impact on the protein (SIFT prediction: deleterious, PolyPhen-2 prediction: possibly damaging, AlphaMissense prediction: likely pathogenic). Additionally, CB1 is the main cannabinoid receptor in the brain, and this variant is located on an important helix responsible for receptor flexibility and movement [23].

**Fig 3:**
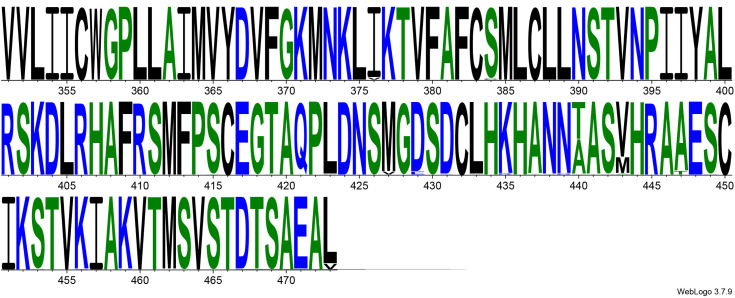
Multiple sequence alignment of CB1 receptor orthologs from 86 species, visualized using WebLogo (the figure is focused on the last 122 amino acids). Blue represents hydrophilic residues, green represents neutral residues, and black represents hydrophobic residues.

From the Blastp analysis of the CB1 amino acid sequence, the top 100 sequences were considered. A filtration process was applied to remove low-quality sequences and predicted sequences, while retaining a single isoform per species, resulting in a set of 86 sequences. The final dataset (S2 Supplementary) demonstrated exceptional sequence integrity, with 100% query coverage and percent identity ranging from 95.55% to 100% across all species. The alignment was conducted using Clustal Omega, and the resulting alignment was uploaded to WebLogo, which gave a visual illustration of the 100% conservation of the amino acid V in position 392 on the CB1 sequence (position 393 on the alignment figure) (Fig 3).

The quality of the newly modelled CB1 structure, using Schrodinger Prime, was evaluated via its Ramachandran plot. Over 90% of protein residues fell within the most favored regions, and 7.9% within the additional allowed regions (S3 Supplementary, Figure 1), indicating its reliability for further computational studies. Then, the variant V392A (MT) was generated by inducing a residue mutation in Maestro.

After the structural preparation and refinement of the 3D models for both the WT and MT proteins, MD simulations were performed to analyze the fluctuations of the macromolecule. This approach aimed to identify key conformational changes occurring in the protein following the mutation, providing insight into the molecular basis of the predicted pathogenicity of the mutant protein. To eliminate random variability and enhance the reliability of the results, three independent replicates were conducted for each model using different random seeds.

**Fig 4:**
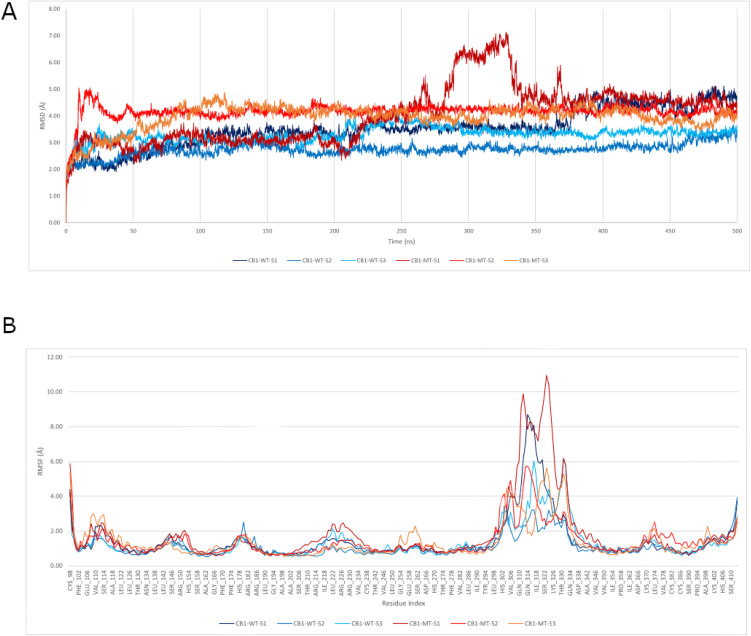
Plots of structural fluctuations of CB1-WT and CB1-MT over the 500 ns MD simulation trajectories. (A) RMSD and (B) RMSF. The blue shades correspond to the WT replicas, while the red ones correspond to the MT replicas. Values are in Angstroms (Å).

The MD simulation trajectories reveal a clear difference in structural fluctuations between WT and MT CB1 proteins over time. RMSD analysis, a key metric for evaluating structural changes, shows that WT CB1 in all seeds is more stable than MT CB1 (Table 3, Fig 4-A). This suggests that the V392A mutation increases the protein’s flexibility, potentially impacting the receptor’s structural integrity.

**Table 3:** The averages and standard deviation (Avg ± SD) of RMSD and RMSF calculated for MD simulation trajectories of the six simulations for the WT-CB1 and MT-CB1.

Parameters	Analyzed structures					
	CB1-WT-S1	CB1-WT-S2	CB1-WT-S3	CB1-MT-S1	CB1-MT-S2	CB1-MT-S3
RMSD (Å)	3.52 ± 0.71	2.74 ± 0.25	3.30 ± 0.29	4.02 ± 1.08	4.16 ± 0.28	3.95 ± 0.47
RMSF (Å)	1.44 ± 1.27	1.12 ± 0.63	1.35 ± 0.94	1.78 ± 1.85	1.42 ± 0.92	1.40 ± 0.98

This increased fluctuation is further highlighted by RMSF analysis, which reflects the flexibility of Cα atoms per residue. Most residues in the three WT protein simulations exhibit lower RMSF values compared to the MT (Table 3, Fig 4-B).

Additional analysis of SSE revealed that all the MT have lost a percentage of their helices (Table 4), further demonstrating the destabilizing effect of this variant. All three WT timelines show strong maintenance of overall secondary structure, with the %SSE consistently high (typically above 66%) and little fluctuation throughout the entire 500 ns simulation. This indicates robust architectural stability of the wild-type CB1 receptor (S3 Supplementary, Figure 2). The continuous horizontal red bands highlight persistent helicity in major structural regions. Short interruptions or breaks in the bands correspond to local flexibilities, commonly observed in loops or edge residues, but are relatively sparse and do not propagate over time. Across all WT replicates, these interruptions are limited, and there is no evidence of large-scale loss or propagation of disorder, which speaks to the energetic favorability of the native fold. There is minimal evidence of transient or persistent local unfolding, even under stochastic variation from different seeds, further supporting the resilience of the wild-type fold.

**Table 4:** SSE percentage (%) calculated over the 500 ns MD simulation trajectories.

	CB1-WT	CB1-MT
S1	69.41%	65.40%
S2	68.46%	66.73%
S3	66.87%	64.77%

In the MT replicates, while the overall %SSE remains relatively high (>60%), per seed comparison demonstrates a consistent loss rate (4.01%, 1.73%, and 2.10% for seeds S1, S2, and S3 Supplementary respectively). The increased jaggedness and more noticeable excursions away from the WT mean %SSE are a direct signature of decreased structural stability.

Across all three MT SSE timelines, the horizontal red bands are more interrupted, and white gaps are more frequent, sometimes spanning longer time windows, which highlights a greater tendency for specific helices to lose their structure transiently. Certain segments (as visualized by clearer gaps in red horizontal stripes) show repeated loss and partial regaining of secondary structure, suggesting structural lability or partial unfolding, especially in regions surrounding the mutation site (between index 275 and 300) (Supplementary 3 Fig 3).

While some regions of increased disorder and lability are reproducible across all MT runs, there is also variability in the extent and timing of structural loss depending on the seed. This is typical for destabilized proteins that sample a broader range of conformational states. Nonetheless, the pattern of increased disorder is present in all three MT seeds, supporting a robust mutational effect.

**Fig 5:**
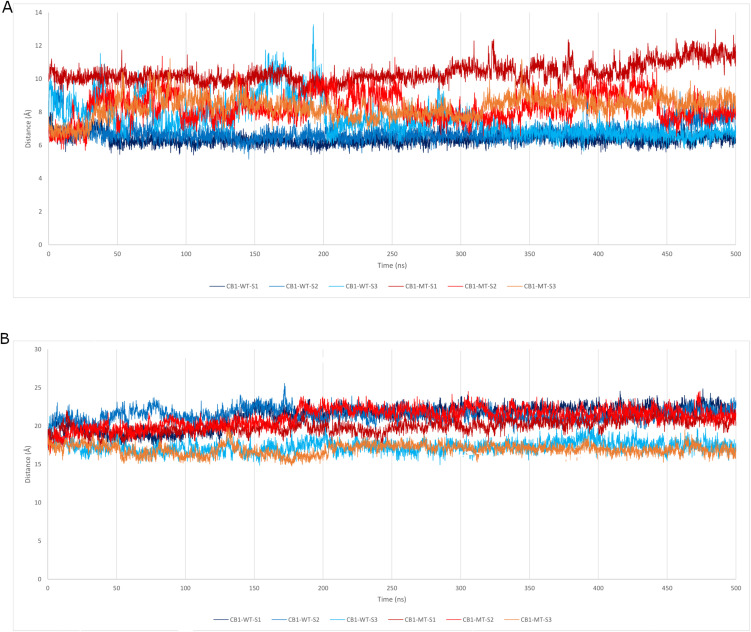
Plots of the fluctuations of the distance between (A) TM3 and TM6 and (B) TM3 and TM7 in CB1-WT and CB1-MT over simulation trajectories, in Angstroms (Å). The blue shades correspond to the WT replicas, while the red ones correspond to the MT replicas.

The TM3-TM6 and TM3-TM7 distances show an interesting conformational change within the protein, due to the mutation. WT models show TM3-TM6 distances clustering tightly in the 6.4–7.6 Å range, with minimal drift or fluctuation throughout (SDs 0.35–1.07). While MT replicas exhibit greater and consistently elevated TM3-TM6 distances (8.29–10.35 Å, SDs 0.60–0.84, specifically MT-S1, which shows the highest distance increase (Fig 5-A) (Table 5).

**Table 5:** The averages and standard deviation (Avg ± SD) of the distances between TM3-TM6 and TM3-TM7, calculated for MD simulation trajectories of the six simulations for the WT-CB1 and MT-CB1.

Parameters	Analyzed structures					
	CB1-WT-S1	CB1-WT-S2	CB1-WT-S3	CB1-MT-S1	CB1-MT-S2	CB1-MT-S3
TM3-TM6 distance (Å)	6.37 ± 0.35	6.77 ± 0.53	7.60 ± 1.07	10.35 ± 0.60	8.29 ± 0.84	8.30 ± 0.66
TM3-TM7 distance (Å)	21.30 ± 1.43	21.44 ± 0.84	17.31 ± 0.79	19.98 ± 0.86	21.15 ± 1.27	16.92 ± 0.71

The WT TM3-TM7 distances are generally larger (Fig 5-B), indicating a stable, open conformation compatible with inactive receptor fold. But MT replicas show lower distances.

The intramolecular H-Bond analysis revealed that the MTs tend to have fewer H-Bonds than the WTs. This further explains the elevated RMSD and RMSF values, and could also offer a possible explanation for the shift in the monitored interhelical distances (Table 6).

**Table 6:** The averages and standard deviation (Avg ± SD) of the intramolecular H-Bonds, calculated for MD simulation trajectories of the six simulations for the WT-CB1 and MT-CB1.

Parameters	Analyzed structures					
	CB1-WT-S1	CB1-WT-S2	CB1-WT-S3	CB1-MT-S1	CB1-MT-S2	CB1-MT-S3
H-Bond	329.31 ± 7.38	325.51 ± 7.69	319.48 ± 7.48	326.45 ± 7.47	317.86 ± 8.28	317.28 ± 7.54

The FEL was constructed using RMSD and Rg as joint collective variables to provide a rigorous thermodynamic mapping of the conformational space.

**Fig 6:**
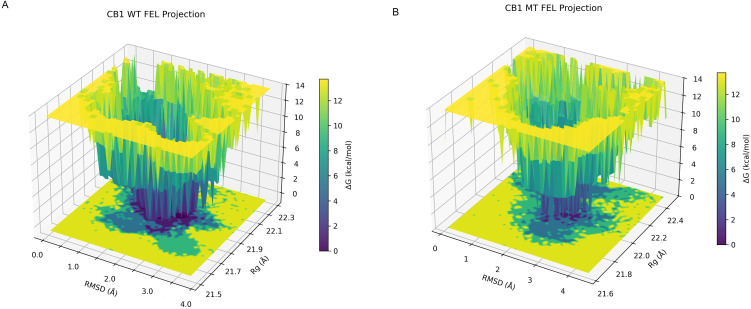
3D FEL projections for WT (A) and MT (B) trajectories, showing conformational stability and energetic states as a function of RMSD and radius of gyration (Rg).

The FEL analysis of WT trajectories shows a deep and narrow basin at RMSD values between 1.5 and 2.5 Å, and Rg values between 21.9 and 22.1 Å (Fig 6-A). This sharp, well-defined minimum corresponds to stable and compact conformational states, with a steep surrounding energy barrier, indicating low structural fluctuation and a dominant global minimum.

The FEL analysis of MT trajectories shows a broader and flatter basin, with multiple accessible minima at higher RMSD and a slightly expanded Rg range. The main basin is shifted towards higher RMSD values (2.5 to 3.5 Å) and more diffuse Rg (21.0–22.4 Å), and the lowest minimum is less deep compared to WT (Fig 6-B). This highlights a broader and more elevated energy plateau.

**Fig 7:**
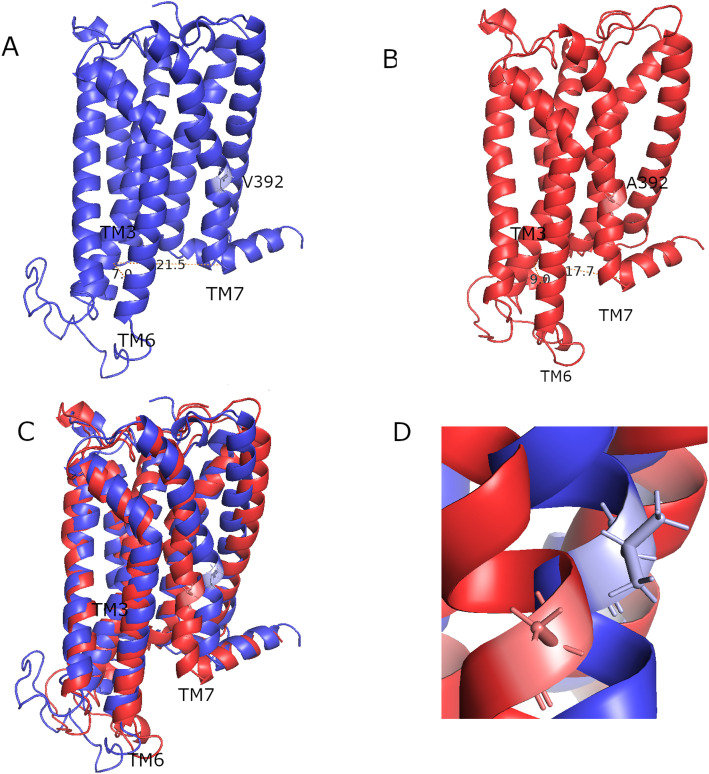
Representative conformations and helical rearrangements in(A) the WT CB1 receptor (blue), (B) the V392A MT CB1 receptor (red), (C) Superimposition of WT and MT aligned to the conserved transmembrane core. Arrows indicate the outward flare of TM6 (increased TM3–TM6 distance) and the inward shift of TM7 (decreased TM3–TM7 distance), and (D) detailed view of the MT amino acid (Alanine) and WT amino acid (Valine) site.

The comparison of the energy distribution of all MT trajectories to the WT trajectories shows that the MTs’ free energy appears higher than that of the WTs. This provides quantitative backing that the V392A mutation raises the free energy barrier for occupancy of the stable, native-like structure. The most representative conformations extracted from the global energy minima of both the WT and MT landscapes further display the distinct conformational divergence between the WT and V392A MT systems (Fig 7-A and B). By anchoring the alignment on the conserved transmembrane (TM) core (Fig 7-C), we identified a coordinated shift in the intracellular helical arrangement. In the MT receptor, the TM6 intracellular segment exhibited a slight outward displacement, paired with an inward movement of the TM7 intracellular segment.

## 4. Discussion

The comprehensive genomic analysis of eleven key endocannabinoid system (ECS) genes in a cohort of 109 Moroccan individuals reveals a rich landscape of genetic variation with important biomedical implications [18]. Among the 7,415 variants identified, modifier and low-impact variants overwhelmingly predominate. However, several variants identified in genes such as *MGLL, ABHD12 FAAH, and DAGLA* are particularly noteworthy given their documented association with human disorders, including metabolic, neuropsychiatric, and neurodevelopmental conditions.

*MGLL* is a gene that codes for the protein MGLL (also known as MAGL), which is the main enzyme responsible for the degradation of 2-AG via hydrolysis. It is localized presynaptically and accounts for 80% of the total 2-AG degradation activity within the ECS [15,33,34]. Mutations in MGLL have been linked to metabolic syndromes, obesity, and neurodegenerative diseases due to dysregulated endocannabinoid signaling affecting inflammation and neuronal homeostasis. Our data revealed the presence of three MGLL variants, which have been linked to MGLL-related disorder [35].

ABHD12 also hydrolyses 2-AG, as it is responsible for 9% of the total 2-AG degradation activity [17,36]. Additionally, mutations in ABHD12 cause PHARC syndrome (polyneuropathy, hearing loss, ataxia, retinitis pigmentosa, and cataract), illustrating the significance of ECS integrity in maintaining neural health [37]. Our data highlight the presence of seven ABHD12 variants in the study population, which have previously been linked to this syndrome [38].

Variants in FAAH, the main enzyme for anandamide catabolism, are associated with alterations in pain sensitivity, anxiety, and a predisposition to substance abuse, exposing their vital role in modulating cannabinoid receptor function and neurobehavioral outcomes [14,15]. Our data shows 2 variants linked to FAAH-related disorder, mainly responsible for susceptibility to drug addiction [39].

DAGLA or diacylglycerol lipase alpha, the principal enzyme for synthesizing 2-AG in the central nervous system. 2-AG serves as the most abundant endocannabinoid and a primary ligand for CB1 receptors, critically modulating retrograde neurotransmission, neurogenesis, and synaptic plasticity [40,41]. Rare DAGLA variants have been associated with seizures, neurodevelopmental disorders, and abnormalities in brain morphology. Loss of DAGLA function in animal models leads to impaired adult neurogenesis and anxiety-like behaviors, underscoring its essential role in neural development and function. The presence of pathogenic and novel DAGLA variants in the Moroccan dataset suggests potential implications for neurological disease susceptibility in this population [42–44].

The identification of 170 novel variants within all the genes implicated in the ECS broadens the catalog of the Moroccan ECS genetic diversity, providing a valuable resource for functional genomics and precision medicine efforts in North African populations. The detection of 43 novel missense mutations within the exonic regions of the genes, with four classified as pathogenic by multiple predictive algorithms, underscores the functional importance of these variants.

Particular focus, within our framework, has been allocated to the novel variant V392A, located on the *CNR1* gene. It encodes for the GPCR CB1 and has long been studied as it is the main receptor responsible for THC’s psychotropic effect on the brain [1,45].

The computational investigation conducted in this study revealed profound effects of the V392A mutation, localized to TM7 of the CB1 receptor, on its structural dynamics and conformational stability. The CB1 receptor is abundant in the human brain cortex, specifically within the association and limbic cortices, and at lower levels in the motor and primary sensory regions. They are also located presynaptically in gamma-aminobutyric acid-ergic neurons and the hippocampus [46]. Its TM7 is known to play a critical role in defining the conformational states of GPCRs by contributing to ligand binding and coupling with G-proteins and β-arrestins. Mutation-induced perturbations in this helix can therefore have widespread functional consequences [47].

Compared to the WT receptor, all MT replicas showed increased backbone deviation (RMSD) and elevated atomic fluctuations (RMSF), particularly proximal to the TM7 region harboring the mutation. This increased flexibility likely arises from the disruption of intrahelical compactness of the amino acids involved in this region, which is essential for maintaining TM7’s structural integrity. All MT replicates exhibited increased backbone deviation (RMSD) and elevated atomic fluctuations (RMSF), particularly in the regions proximal to the TM7 domain harboring the mutation. This heightened flexibility likely originates from the disruption of the intrahelical packing and local interaction network, which is essential for maintaining the structural integrity of TM7. This is supported by our intramolecular hydrogen bonding analysis, as MTs consistently exhibited a lower average number of bonds compared to the WTs. This localized destabilization propagates to neighboring helices, notably TM3 and TM6, as evidenced by the altered interhelical distances and the shifts observed in the Free Energy Landscapes.

Notably, the TM3-TM6 distance significantly increased in the MT trajectory ensemble, reflecting a disruption in the helical packing that is crucial for receptor activation dynamics. TM6 outward movement is a hallmark of GPCR activation [48], and its uncoupling from TM3 due to TM7 mutation suggests impaired allosteric communication along the helix bundle TM3-TM6-TM7 [49,50]. Situated immediately proximal to the conserved NPxxY activation microswitch, the substitution of Valine for the smaller Alanine creates a packing defect that triggers an inward shift of TM7. This rearrangement, combined with a systematic reduction in intramolecular hydrogen bond occupancy, uncouples the intracellular end of TM6. The resulting outward shift of TM6 signifies a transition toward an active-like state, providing a mechanistic link between this Moroccan-specific variant and altered signaling.

This induced an active-like conformation characterized by a larger TM3-TM6 distance compared to WT simulations. Concurrently, the MTs’ TM3-TM7 distance decreased, consistent with local compression or misalignment around the mutation site that may hinder conformational transitions necessary for signaling. GPCR TM7 displayed a clear inward movement, hinting at a receptor activation [23].

Secondary structure element analysis further confirmed the loss of structural integrity within TM7, as evidenced by a consistent reduction in α-helicity within TM7 in MT runs, correlating with the mutation site, and an increase in transient helical disruptions across the receptor. These local secondary structure perturbations contribute to the observed increase in conformational heterogeneity and energetic destabilization.

FEL projections strikingly capture these mutational effects; the MT FEL is broadened with multiple shallow minima displaced to higher RMSD and Rg values compared to the WT, which displays a narrow, deep energy basin consistent with a well-folded, stable receptor conformation. This shift toward a more diverse and less stable conformational ensemble suggests that the V392A mutation raises the energetic cost of adopting native-like states, weakening overall receptor stability and making aberrant conformations more accessible.

The significance of the V392 residue is further supported by existing genomic data. According to the UniProt database (Accession: P21554), two other substitutions at this position, V392M and V392E, have been recorded. Although their clinical significance remains categorized as uncertain, they are computationally predicted to be deleterious [51]. Our discovery of the V392A variant in the Moroccan population adds a new dimension to this site’s mutational landscape. The consistent prediction of impairment across different amino acid substitutions (Methionine, Glutamate, and now Alanine) strongly suggests that the Valine side chain at position 392 is structurally essential for maintaining the local hydrophobic packing and the overall integrity of the CB1 receptor.

Collectively, these findings highlight the critical role of TM7’s structural integrity in maintaining CB1 receptor function and underscore how mutations in this domain induce destabilization at multiple levels, including local secondary structure, interhelical packing, and global conformational energy landscapes. Such disruptions lead to the deviation of the receptor from its inactive state, likely impairing ligand binding, G-protein coupling, and receptor responsiveness, offering a mechanistic rationale for potential altered pharmacological responses associated with this mutation.

While our results are based on robust computational data, we recognize the importance of experimental confirmation. This study provides a strong mechanistic hypothesis that we plan to validate through future *in vitro* work, including site-directed mutagenesis and cAMP accumulation assays to definitively confirm how the V392A variant impacts CB1 receptor downstream signaling and G-protein coupling.

## Conclusion

This study provides valuable insights into the genetic architecture of the Moroccan population’s ECS, emphasizing the need for population-specific investigations to guide precision medicine and public health strategies in North Africa. Computational structural analysis revealed the functional significance of a highly conserved novel CB1 mutation, underscoring how region-specific ECS genetic diversity may influence cannabinoid response, disease susceptibility, and personalized therapeutic options. The discovery of novel variants represents a valuable resource for future studies assessing their functional impact on endogenous and exogenous cannabinoid ligand interactions. Overall, integrating population genomics with molecular biophysics advances understanding of cannabinoid receptor biology and supports the development of tailored neuropharmacological interventions in genetically diverse populations.

## Supporting information

S1 Supplementary The full list of all novel variants identified in this study. (CSV)

S2 Supplementary Summary of CNR1 sequences resulting from BLASTp filtration. (CSV)

S3 Supplementary Figure 1 representing the Ramachandran plot of the modelled CB1 protein structure, showing backbone dihedral angle distribution and overall stereochemical quality assessment, Figure 2 representing the SSE evolution over time of the WT structures during 3 replicas of MD simulations, and Figure 3 representing the SSE evolution over time of the MT structures during 3 replicas of MD simulations. (DOCX)
