# Supplementary material for: Genomic and structural analysis of the endocannabinoid system in Moroccans: A novel CNR1 variant (V392A) impairs CB1 receptor stability
Source: PLoS One. 2026 Apr 30;21(4):e0347606. doi: 10.1371/journal.pone.0347606 (PMC13132444; doi:10.1371/journal.pone.0347606)

# Supplementary Data


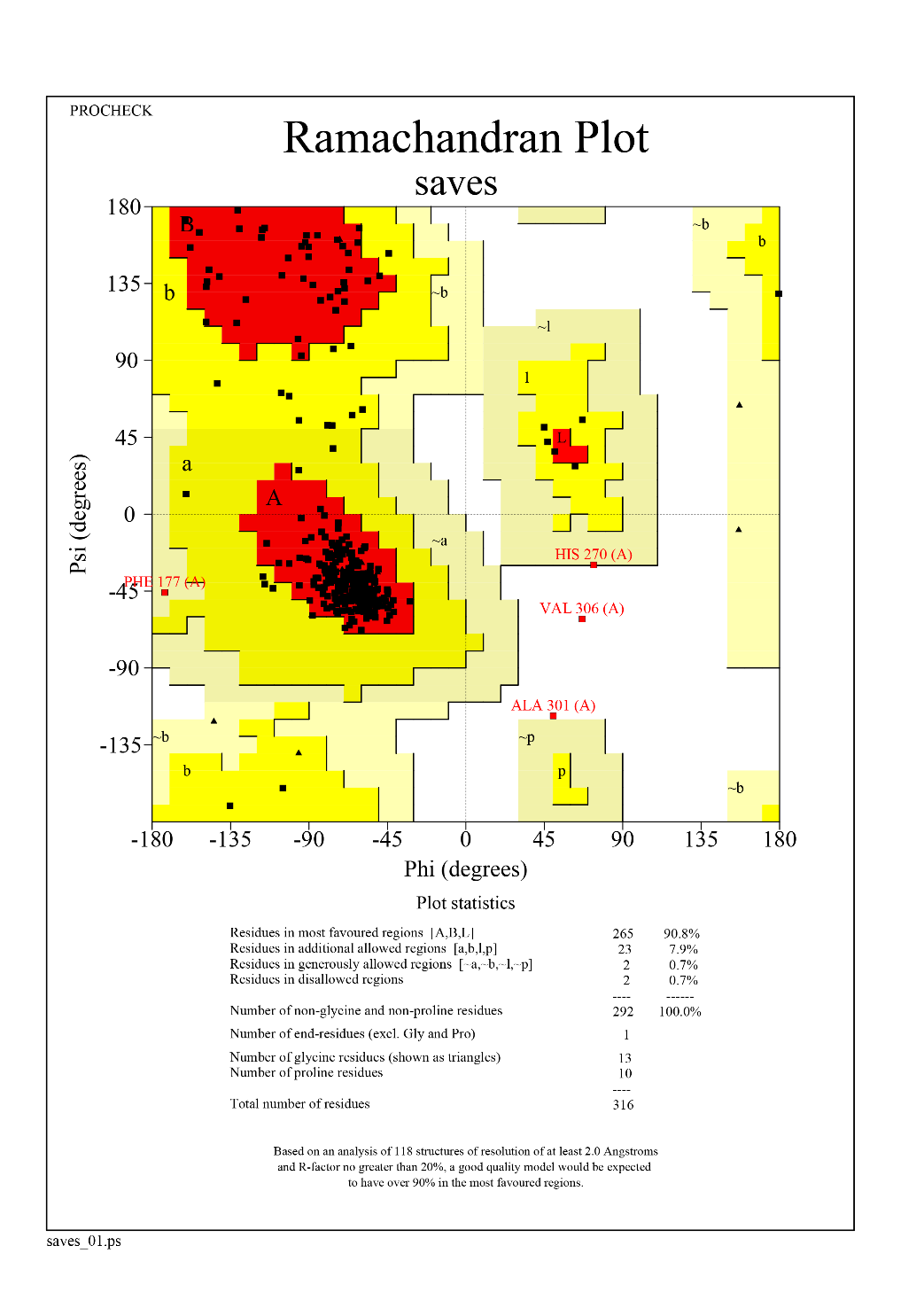
Figure 1: Ramachandran plot of the modelled CB1 protein structure, showing backbone dihedral angle distribution and overall stereochemical quality assessment.

Figure 2: SSE evolution over time of the WT structures during 3 replicas of MD simulations.


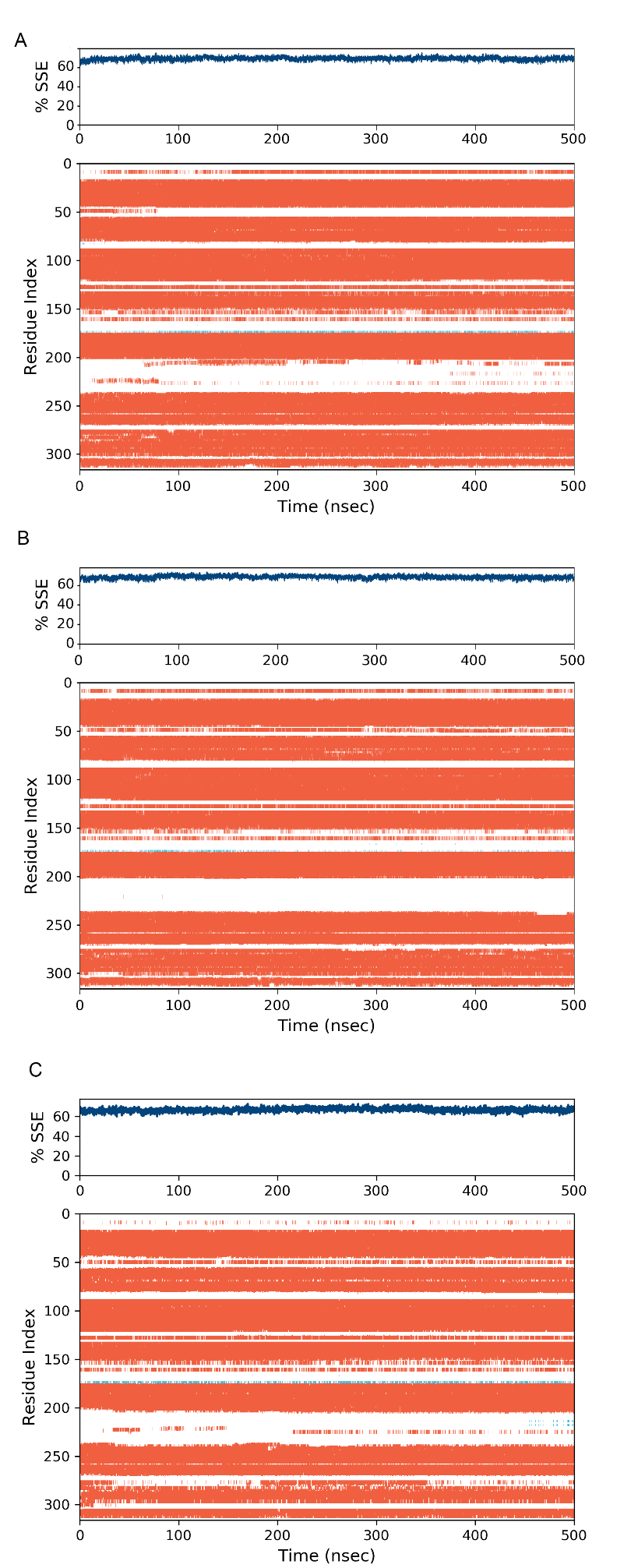


Figure 3: SSE evolution over time of the MT structures during 3 replicas of MD simulations.


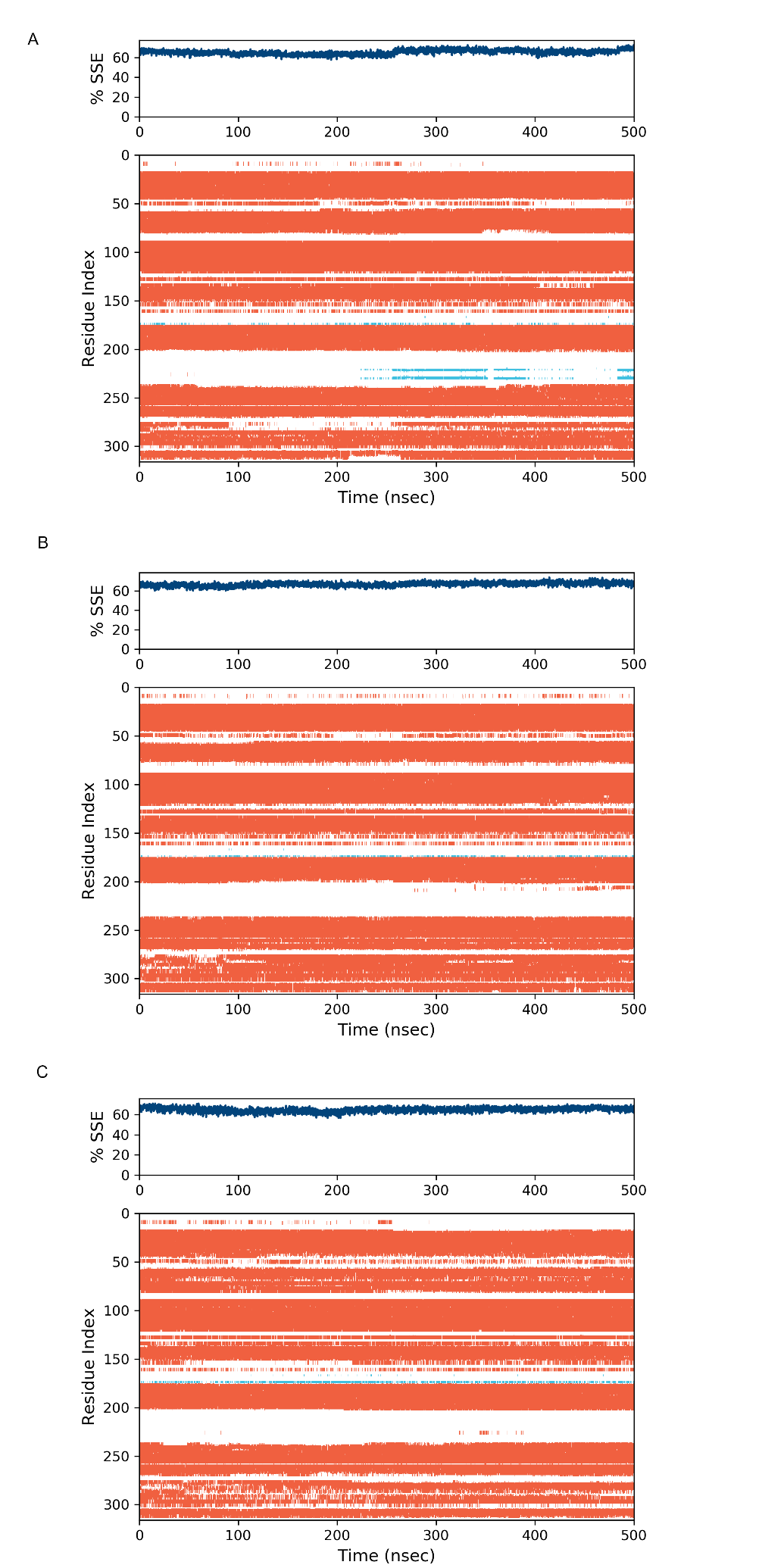

Supplement: S3 Supplementary — (DOCX) [file pone.0347606.s003.docx]
